# 364. Individualized Prognostics in COVID-19 Facilitated by Computer Recognition of Blood Leukocyte Subsets

**DOI:** 10.1093/ofid/ofab466.565

**Published:** 2021-12-04

**Authors:** Claudia R Libertin, Prakash Kempaiah, Ravindra Durvasula, Ariel Rivas

**Affiliations:** 1 Mayo Clinic, Jacksonville, Jacksonville, FL; 2 Mayo Clinic, Jacksonville, Florida; 3 University of New Mexico, Albuquerque, New Mexico

## Abstract

**Background:**

To determine whether CBC differentials of COVID+ inpatients can predict, at admission, both maximum oxygen requirements (MOR) and 30-day mortality.

**Methods:**

Based on an approved IRB protocol, CBC differentials from the first 3 days of hospitalization of 12 SARS CoV-2 infected patients were retrospectively extracted from hospital records and analyzed with a privately owned Pattern Recognition Software (PRS, US Patent 10,429,389 B2) previously validated in sepsis, HIV, and hantavirus infections. PRS partitions the data into subsets immunologically dissimilar from one another, although internally similar.

**Results:**

Regardless of the angle considered, the classic analysis −which measured the percentages of lymphocytes, monocytes, and neutrophils− did not distinguish outcomes (A). In contrast, non-overlapping patterns generated by the PRS differentiated 3 (left, vertical, and right) groups of patients (B). One subset was only composed of survivors (B). The remaining subsets included the highest oxygenation requirements (B). At least two immunologically interpretable, multi-cellular indicators distinguished the 3 data subsets with statistically significant differences (C, *p*≤ 0.05). Survivors (the left subset) showed lower N/L and/or higher M/L ratios than non-survivors (the vertical subset, C).Therefore, PRS partitioned the data into subsets that displayed both biological and significant differences. Because it offers visually explicit information, clinicians do not require a specialized training to interpret PRS-generated results.

CBCs vs. outcomes - Software-analyzed CBCs vs. outcomes

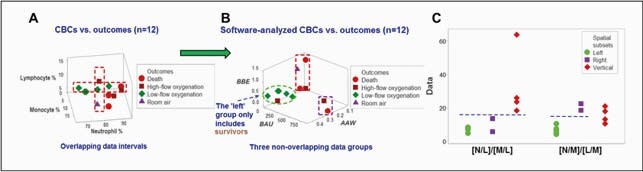

**Conclusion:**

(1) Analysis of blood leukocyte data predicts MOR and 30-d mortality. (2) Real time PRS analysis facilitates personalized medical decisions. (3) PRS measures two dimensions rarely assessed: multi-cellularity and dynamics. (4) Even with very small datasets, PRS may achieve statistical significance. (5) Larger COVID+ infected cohort is being analyzed for potential commercialization.

**Disclosures:**

**Claudia R. Libertin, MD**, **Gilead** (Grant/Research Support)

